# Color density spectral array for early evaluation of prognosis of patients with coma in pediatric intensive care unit

**DOI:** 10.1097/MD.0000000000017493

**Published:** 2019-10-11

**Authors:** Jiangtao Wang, Xiaosheng Hao, Yanfeng Zhang, Guiling Liu, Yinbo Chen, Ge Qu, Chunnv Li, Jianmin Liang

**Affiliations:** a Department of Pediatric Neurology; b Research center of Neuroscience, Bethune First Hospital of Jilin University, Changchun China.

**Keywords:** color density spectral array, coma, electroencephalogram, pediatric patient, prognosis

## Abstract

The aim of this study was to assess the bedside brain function monitoring of color density spectral array (CDSA) for early prognostic evaluation of coma patients in pediatric intensive care unit (PICU).

Forty-two consecutive pediatric coma patients were enrolled. The individual conscious state was evaluated according to the Glasgow coma scale (GCS). CDSA parameters including CDSA pattern (CDSAP), sleep–wake cycle (SWC), sleep stage (SS), and drug-induced fast wave activity (DIFWA) were recorded. Three months later, prognosis was evaluated according to pediatric cerebral performance category (PCPC) score, based on which the patients were divided into FP-group (favorable prognosis) and PP-group (poor prognosis).

The changeable type of CDSAP, appearance of SWC, SS, and DIFWA were significantly correlated with favorable prognosis. Both GCS and SWC were significantly correlated with the prognosis. However, there was substantial overlap in GCS between FP-group and PP-group. Although the absence of SWC was statistically an independent risk factor for poor prognosis but with a high false positive rate (0.143), a linear logistic regression showed the odds ratio of GCS for predicting prognosis was 0.93 (95% confidence interval: 0.48–1.80; *P* = .83) and that of SWC was 0.12 (95% confidence interval: 0.03–0.47; *P* = .03). Furthermore, the absence of SWC was correlated with poor prognosis in nonintracranial infection patients.

Our study found that several CDSA factors are associated with prognosis of coma patients in PICU. SWC may be a potential indicator for evaluating the prognosis of coma patients in PICU.

## 1 Introduction

Coma is a severe condition in PICU, and bedside brain function monitoring is a crucial method for prognostic evaluation. Currently, the Glasgow coma scale (GCS) is the most widely used tool for evaluating the conscious state and brain functions. The 15-point scales include 3 aspects including eye opening response, verbal response, and motor response. A lower GCS score indicates a more severe condition and predicts a poor prognosis. However, the GCS scoring system has some inherent limitations: drug use or low blood oxygen can alter the patient's level of consciousness, leading to an inaccurate score on the GCS; tracheal intubation or tracheotomy and severe eye swelling may make it impossible to test the verbal responses and eye opening; and the assessment of these responses has a certain of subjectivity. Thus, an objective evaluation method is needed for bedside brain function monitoring and prognosis prediction.

Electroencephalography (EEG) is a noninvasive electrophysiological monitoring method for recording the spontaneous electrical activity of the brain, which can sensitively reflect brain injury and the metabolism of cerebral neuronal cells. The amplitude in EEG decreases when the neurons are injured or suppressed, and abnormal metabolism or reduced conduction velocity in nerve fibers can lead to a reduced frequency.^[1]^ However, conventional continuous EEG monitoring is information-heavy, time-consuming, and hard to analyze, and thus, it is rarely used bedside in PICU. Color density spectral array (CDSA) is a quantitative and simplified EEG method that uses Fourier transformation to reflect EEG signals by color and frequency (*y*-axis) over time (*x*-axis).^[2]^ The interpretation of CDSA is much more intuitionistic and easier, and this technique has been applied in critically ill patients.^[3,4]^ Nevertheless, the prognostic value of CDSA remains unclear. In this study, we aimed to investigate the clinical value of CDSA for early evaluation of prognosis in patients with coma in PICU.

## 2 Materials and methods

### 2.1 Patients

A total of 42 consecutive pediatric patients with coma in PICU in the First Hospital of Jilin University were enrolled between March 2016 and March 2017. This study was approved by the local Institutional Review Board and Ethics Committee. Written informed consent was obtained from the guardians of each pediatric patient. Coma was determined according to the GCS.^[5]^ The inclusion criteria were coma within 3 days; GCS ≤8; and age ranging from 1 to 14 years. The exclusion criteria included coma induced by antiepileptic drugs or sedative drugs; other neurological or psychological disorders that may affect the brain function assessment; or significant artifact in EEG.

### 2.2 Data collection

**Figure 1:**
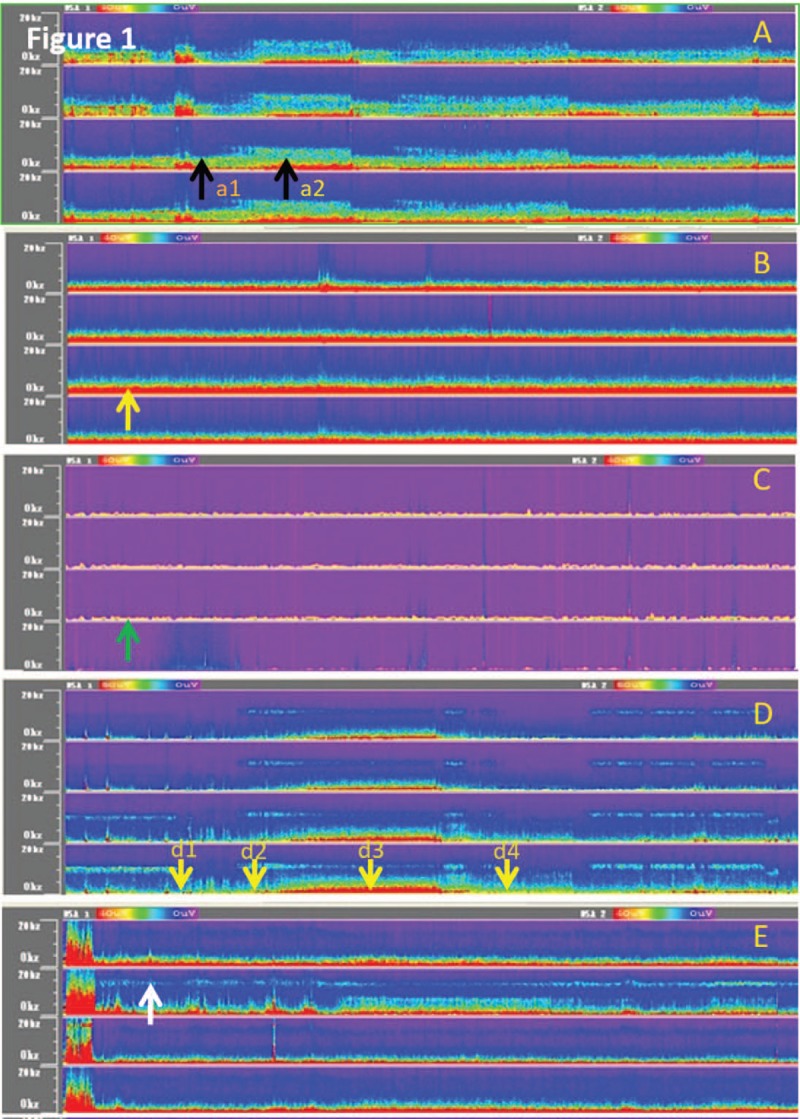
Color density spectral array patterns. (A) Changeable type of color density spectrual arrray patterns (CDSAP) showing higher power spectrum matrixes in the discontinuous high-frequency bands in the context of higher power spectrum matrixes in the low-frequency bands (5–10 Hz, black arrows); *a1* represents the high-power spectrum matrix within the low-frequency band, and *a2* represents the higher power spectrum matrix within the high-frequency band. (B) Slow-monotonous type of CDSAP showing a continuous and invariable spectrum matrix with high power in the low-frequency band (<5 Hz, yellow arrow). (C) Nonspectrum type of CDSAP showing an invariable spectrum matrix with slightly high power, which is thin and close to the baseline (green arrow). (D) Sleep–wake cycle (SWC) showing non-REM and REM sleep stages (SS, d1-3 representing N_1–3_ stages and d4 representing REM stage, see small yellow arrows). (E) Drug-induced fast wave activity (DIFWA) showing a continuous high-power spectrum matrix in the fast-wave band (around 15 Hz, white arrow).

Within 3 days after admission, the individual conscious state was assessed according to the modified GCS.^[6]^ Long-term (≥16 hours) EEG monitoring was performed using a digital EEG monitor (EEG-8102, Digital Versatile Electroencephagraphy, Shenzhen Delica Medical Equipment Co., Ltd., China). The electrodes were placed according to the International 10–20 system, and CDSA of EEG parameters was recorded. The EEG signals in 4 leads (F3/F4 in bilateral frontal areas; O1/O2 in bilateral occipital areas) were transformed into four-channel CDSA patterns (CDSAPs); the spectrum diagram is a two-dimensional image onto which the three-dimensional (time, power, and frequency) images were projected. The *x*-axis represented the time, the *y*-axis represented the frequency, and the color represented the EEG power (power = square of amplitude; μV^2^; high power to low power: red, orange, yellow, green, and blue). The CDSAPs were classified into 3 types according to change of the spectrograms: changeable, which is characterized by a constant and predominant activity in the low-frequency band with variously pronounced and organized peaks in the alpha- or high-frequency band (n = 32; 76.2%; Fig. 1A); slow-monotonous, which is characterized by prominent peaks in the low-frequency band (n = 5; 11.9%; Fig. 1B); and nonspectrum, which is characterized by absence of spectral peak (n = 5; 11.9%; Fig. 1C). The sleep stages (SS) were identified on CDSA: the greatest amount of change in the frequency composition was obtained during spectral analysis, and a succession of relatively stable segments representing stages 2 and 3 of slow sleep as well as REM sleep were identified via modification of the mean power of delta, theta, alpha, and beta activities (Fig. 1D). The fast waves of CDSA were recorded to evaluate the response to sedative drugs (Fig. 1E). One electrophysiologist analyzed the CDSA parameters, including changeable type of CDSAP, sleep–wake cycle (SWC), SS, and drug-induced fast wave activity (DIFWA). The symmetry of background activities between bilateral cerebral hemispheres was not analyzed due to the lack of a standard reference.

Three months after the initial examination, individual prognosis was evaluated according to the pediatric cerebral performance category (PCPC) score (Table 1),^[7]^ which has 6 categories, the patients were divided into such 2 groups: the favorable-prognosis group (FP-group, PCPC 1 to 3 points represent an independent living ability; 13 cases scored 1 point, 5 cases who were able to attend regular school scored 2 points, and 3 cases who received special education scored 3 points), and the poor-prognosis group (PP-group, PCPC 4 to 6 points represent severe disability or death; 9 cases with brain injury who needed other's support scored 4 points, 2 cases with unconsciousness scored 5 points, and 10 died cases scored 6 points).

**Table 1:**
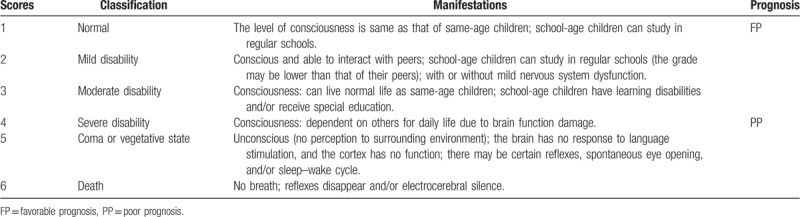
Pediatric cerebral performance category score^[7]^.

### 2.3 Statistical analysis

Statistical analysis was performed using SPSS software package (version 20.0; IBM, Armonk, NY). Continuous variables are presented as mean ± standard deviation (SD); comparison between groups was performed using two-sample *t* test. Chi-squared or Fisher exact test was used to screen the potential risk factors. Wilcoxon rank-sum test was performed to assess the prognostic value of GCS, and Fisher exact test was performed to assess the prognostic value of SWC. Then logistic regression analyses were performed to identify the risk factors for prognosis and to evaluate the prognostic value of combining GCS and SWC. A two-tailed *P* value <.05 was considered indicative of a statistically significant difference.

## 3 Results

### 3.1 Clinical characteristics

The study population included 21 females and 21 males, with an average age of 89.81 ± 46.39 months (range, 16–172 months). According to the PCPC score, the prognosis was favorable in 21 cases (FP-group) and poor in 21 cases (PP-group), yielding a poor prognostic rate of 50%. There were no significant differences in gender, age, and cause of coma between the FP-group and the PP-group (all *P* > .05). The demographic data, the causes of coma, and the etiological comparisons between the FP-group and the PP-group were summarized in Table 2.

**Table 2:**
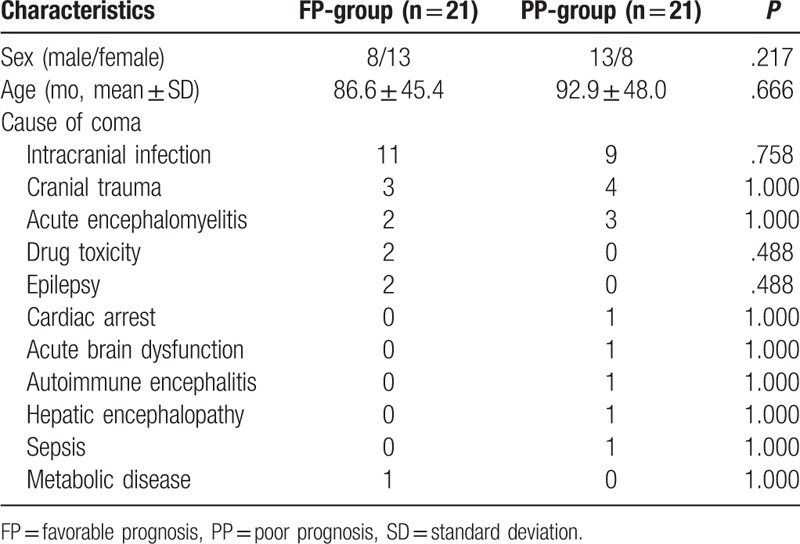
Demographic and etiological comparisons of patients with coma in pediatric intensive care unit.

### 3.2 Prognostic value of GCS

**Figure 2:**
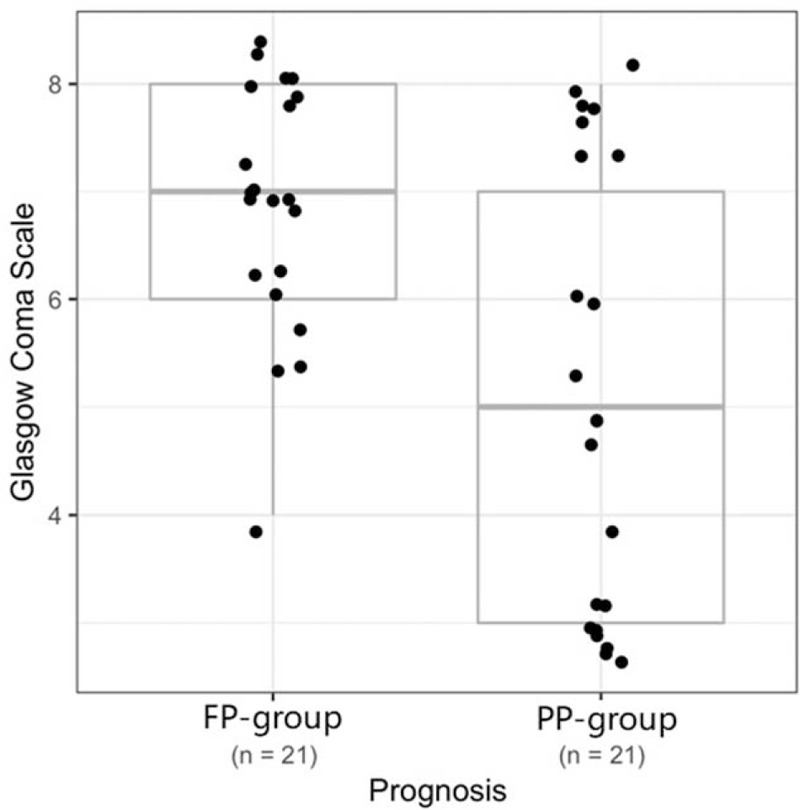
Boxplot of GCS versus prognosis. The FP-group shows higher GCS score than the PP-group (*P* = .01). There is substantial overlap in GCS between these 2 groups, and hence GCS alone cannot serve as a robust indicator of prognosis.

When the 42 children were classified into 3 subgroups according to modified GCS (3–4 points, 5–6 points, or 7–8 points), Fisher exact tests showed GCS was significantly correlated with the prognosis (*P* < .01; see Table 3). The FP-group showed higher GCS score than the PP-group (*P* = .01, Wilcoxon rank-sum test). However, there was substantial overlap in GCS between the FP-group and the PP-group (Fig. 2).

**Table 3:**
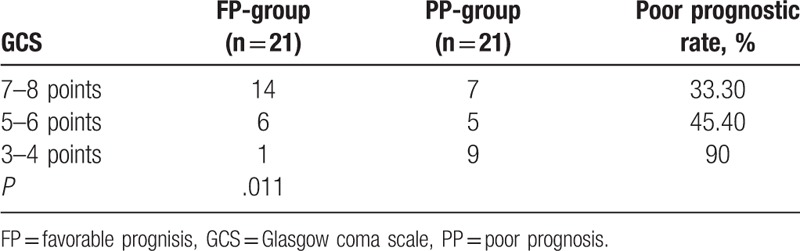
Association between Glasgow coma score and prognosisofpatients with coma in pediatric intensive care unit.

### 3.3 Prognostic value of CDSA

The 42 patients were grouped according to SWC (frequency variation: significant, mild, or absent), SS (sleep spindle waves: frequent, occasional, or absent), DIFWA (present or absent), and CDSAP (changeable type, slow-monotonous type, nonspectrum type). Fisher exact tests showed that changeable type of CDSAP, appearance of SWC, SS, and DIFWA were significantly correlated with favorable prognosis (all *P* < .05; Table 4). Moreover, SWC showed statistical significance in predicting prognosis (*P* = 1.1 × 10^−5^; Table 5). Considering that SWC is closely related to sleeping cycles, 3 factors (SWC, CDSAP, and DIFWA) were included in the subsequent logistic regression analysis, which revealed that the absence of SWC was an independent risk factor predicting poor prognosis (odds ratio [OR] = 43.751; 95% confidence interval [CI] = 2.356–812.463), but a high false positive rate of SWC (0.143 = 1–0.857) was noted. For further analysis, the 42 patients were divided into 2 subgroups: intracranial infection (n = 20) and nonintracranial infection (n = 22). Comparisons of factors including SWC, CDSAP, SS, and DIFWA were conducted between the 2 subgroups within the FP-group and within the PP-group, respectively. Within the PP-group, the absence of SWC was significantly different between the intracranial infection subgroup and the nonintracranial infection subgroup (*P* < .05; Table 6). No other statistical differences were found between the intracranial infection subgroup and the nonintracranial infection subgroup within the FP-group or the PP-group (Table 7).

**Table 4:**
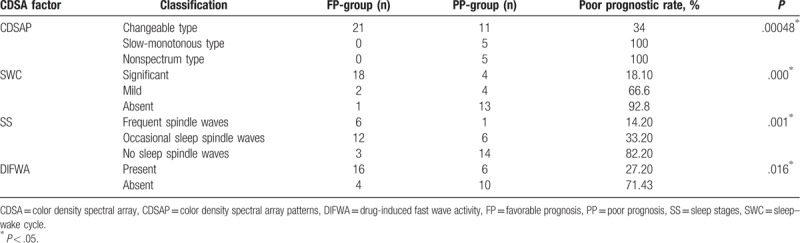
Color density spectral array factors for predicting prognosisofpatients with coma in pediatric intensive care unit.

**Table 5:**
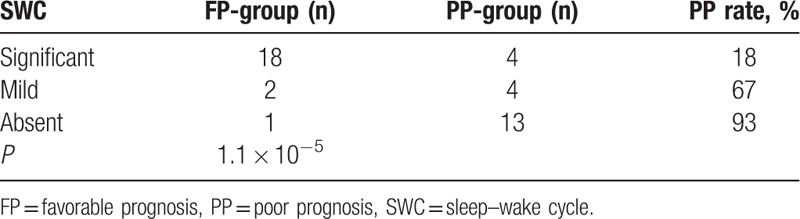
Association between sleep–wake cycle and prognosis of patients with coma in pediatric intensive care unit.

**Table 6:**
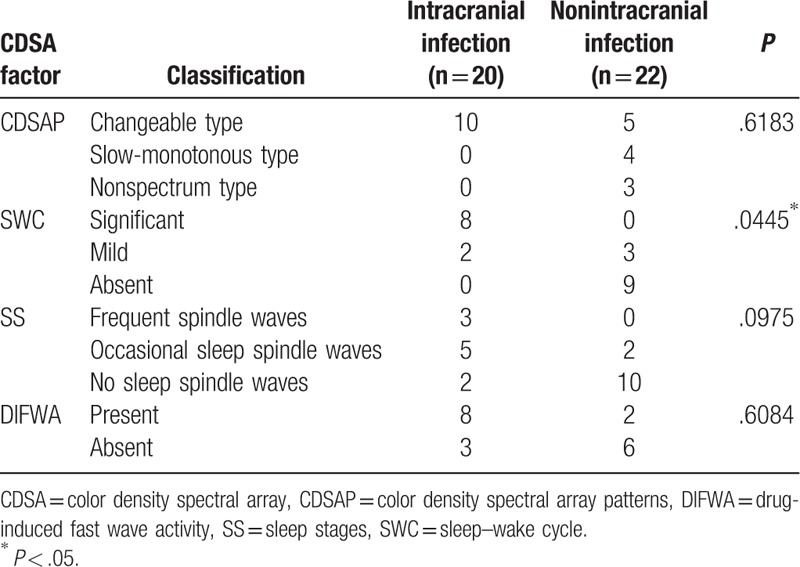
Comparison of color density spectral array factors between intracranial and nonintracranial infection subgroup of poor prognosis patients inpediatric intensive care unit.

**Table 7:**
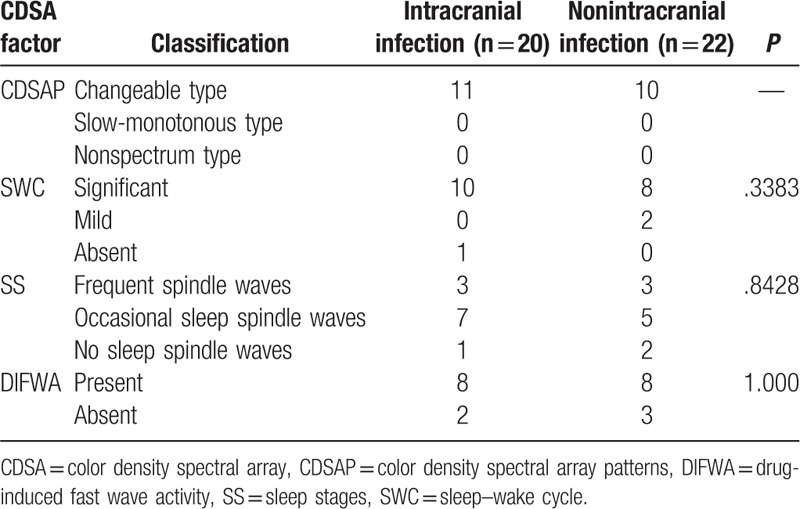
Comparison of color density spectral array factors between intracranial and nonintracranial infection subgroup of favorable prognosis patients in pediatric intensive care unit.

### 3.4 Prognostic value of combining GCS and SWC

As we observed that GCS and SWC might be significantly associated with the prognosis, we further performed a linear logistic regression. The OR of GCS for predicting prognosis was 0.93 (95% CI, 0.48–1.80; *P* = .83), and that of SWC was 0.12 (95% CI, 0.03–0.47; *P* = .03).

### 3.5 Follow-up of CDSA

After a 3-month follow-up, repeated CDSA data were obtained from 19/32 children with changeable type of CDSAP, of which 15 (78.9%) had a favorable prognosis. In addition, among the 5 children with slow-monotonous type, 3 died and 2 remained unchanged CDSAP. Two children with slow-monotonous type of CDSAP had a poor prognosis. All the 5 children with nonspectrum type died.

## 4 Discussion

Brain function monitoring can provide important information for clinical evaluation and prognosis prediction in patients with coma.^[8]^ In current clinical practice, GCS is the most widely used scale for the assessment of conscious status. In the present study, we also noted the correlation between GCS and prognosis, and we found that a lower GCS score predicted a poor prognosis. However, we evaluated the prediction value of GCS, and observed that there was substantial overlap in GCS between FP-group and PP-group, indicating that GCS alone cannot serve as a robust indicator of prognosis in our cohort. The GCS scoring system has some inherent limitations for predicting the prognosis of coma patients in PICU.

Although conventional EEG recording can promote the assessment of conscious status and coma severity, the time-consuming and high analytical skill requirements limit its usage in PICU. CDSA uses Fourier transformation to process long-term EEG signals and converts EEG parameters (such as power, amplitude, and frequency) into visualized images, making it possible to evaluate the dynamic EEG changes in different-frequency bands.^[9]^ Recently, CDSA has been widely used in the clinical evaluation of patients with sleep disorders and epilepsy. However, the application value of CDSA in brain function monitoring has not yet been well elucidated. Here, we examined the reliability of CDSA to predict the prognosis of pediatric patients with coma.

In this study, according to EEG signals, CDSA parameters including CDSAP, SWC, SS, and DIFWA were analyzed. We classified the spectrograms into 3 types: changeable, slow-monotonous, and nonspectrum. On the initial examination, 32 children (76.1%) exhibited changeable type, of which 21 had a favorable prognosis. Among the other 11 children with poor prognosis, only 4 children had significant SWC (36.4%), and these 11 children did not show DIFWA. Five children showed slow-monotonous type and 5 children showed nonspectrum type. CDSA of these 10 children had no SWC, SS, or DIFWA, and 8 of these children died (the other 2 children remained functionally disabled). These findings indicate that changeable type of CDSAP may predict a better prognosis than slow-monotonous type and nonspectrum type, which is consistent with the findings reported by Bricolo et al.^[10]^ We speculate that the brain may still reserve certain adjustment function in the early period of coma, manifesting as diffuse slow waves on EEG, whereas by 3 to 5 days after coma onset, the brain function may deteriorate due to abnormal circulation and metabolism. Furthermore, the follow-up data showed that slow-monotonous type and nonspectrum type of CDSAP were associated with a poor prognosis. Changeable type may indicate that a child's brain function impairment is still reversible, and early effective treatment may help the function recovery.

There were 22 children had clear SWC, all of which showed changeable type, and 18 (81.8%) children among them had a favorable prognosis. Notably, these 18 children had power variation or a fast wave spectrum in the 12 to 14 Hz frequency band, yielding identifiable SS. Of 22 children with SWC, 4 had a poor prognosis (all of them had no SS or DIFWA). Of 14 children without clear SWC, 13 (92.9%) had poor prognosis and 1 (7.1%) remained functionally disabled. Ayas et al found that sleep deprivation may adversely affect the metabolic, immune, and circulatory functions, and they also proposed that sleep disorder can interfere the nerve regeneration.^[11]^ Thus, the preservation of physiological sleep structure may be closely associated with a favorable prognosis. Dario et al studied 27 patients with subacute disturbance of consciousness and found that the presence of SS can predict a favorable prognosis.^[12]^ Karnaze et al noted that an alternating CDSA pattern was significantly associated with survival in patients with head injuries.^[13]^ Pineda et al found that the sleep structure on EEG was correlated with early prognosis of acute encephalopathy and the presence of SS could predict favorable outcomes.^[14]^ In the present study, we used spindle waves to identify the SS. In this study, we used a much larger sample size which supported their speculation.

Considering the presence of the SWC is a prerequisite of SS, we suggest that SWC may be used as a practical and intuitionistic prognostic predictor. Fisher exact test showed that SWC had statistical significance for prognosis prediction. Although logistic regression analysis showed that the absence of SWC was an independent risk factor predicting poor prognosis with a high OR (43.751), SWC was still not a robust indicator to serve as an independent factor to predict prognosis in our cohort, due to the high false positive rate (0.143). As GCS and SWC might be associated with the prognosis, we further performed a linear logistic regression, which indicated that the combination of GCS and SWC could not significantly improve the predictive ability when compared with SWC alone. Therefore, we speculate the prognostic value of SWC in comatose children may be better than that of GCS. Further analysis revealed that the absence of SWC might be a useful predictor for poor prognosis in coma patients without intracranial infection (Table 6). However, in the future studies, we may explore the predictive value of SWC and other potential factors in larger scale cohorts.

The present study has some noticeable limitations. We did not perform repeated monitoring to evaluate the dynamic changes on CDSAP. In addition, the sample size was small, and definitive conclusions still require further studies involving a much larger cohort.

## 5 Conclusion

The CDSA diameters may be a useful bedside tool for PICU physicians to evaluate the status of critical patients. Our study presents some interesting discoveries and found several CDSA factors that may be associated prognosis of coma patients in PICU. Both GCS and SWC were informative but had limitation. The predictive value of SWC is superior to that of GCS, and SWC may be a potential indicator for evaluating the prognosis of coma patients in PICU.

## Acknowledgments

We gratefully acknowledge the valuable cooperation of Prof. Yumei Li, Dr. Cuiying Wang, and the staff who helped us in this study. This work was supported by the National Nature and Science Foundation of China (No. 81771396); the Nature and Science Foundation from the Science and Technology Department of Jilin Province (No. 20180101159JC); and the Special Fund Project of Industrial innovation of Jilin provincial (No. 2017C029-1).

## Author contributions

**Data curation:** Jiangtao Wang, Xiaosheng Hao, Ge Qu, Chunnv Li.

**Formal analysis:** Yanfeng Zhang, Yinbo Chen, Jianmin Liang.

**Methodology:** Ge Qu.

**Resources:** Chunnv Li.

**Validation:** Yanfeng Zhang, Chunnv Li.

**Writing – original draft:** Jiangtao Wang.

**Writing – review and editing:** Xiaosheng Hao, Guiling Liu, Yinbo Chen, Jianmin Liang.
